# A Qualitative Exploration of the Socioecological Influences Shaping the Diagnostic Experience and Self‐Management Practices Among People Newly Diagnosed With Multiple Sclerosis

**DOI:** 10.1111/hex.70091

**Published:** 2024-11-06

**Authors:** Olivia Wills, Sarah Manche, Yasmine Probst

**Affiliations:** ^1^ School of Medical, Indigenous and Health Sciences University of Wollongong Wollongong New South Wales Australia

**Keywords:** lifestyle, lived experience, multiple sclerosis, qualitative, socioecological

## Abstract

**Background:**

People newly diagnosed with multiple sclerosis (MS) often pursue ‘health‐related’ behaviour changes to feel in control of their diagnosis. However, little is known about the specific factors that may influence behaviour change during this crucial time. Therefore, we conducted an in‐depth exploration of the socioecological influences impacting the diagnostic experience and self‐management practices following an MS diagnosis.

**Methods:**

We followed a qualitative study design using a phenomenological approach to explore the lived experiences of people newly diagnosed with MS. Analysis was conducted via an iterative process, starting with deductive open coding to map onto the socioecological model, followed by inductive focused coding to extract key themes from participants' reported experiences.

**Results:**

Eight participants diagnosed with MS within the past 12 months were interviewed. Four themes were reported across the MS journey, reflecting the different levels of the socioecological model: (1) taking control of a new diagnosis to retain a sense of personal identity—individual level; (2) grief and acceptance guided by community—social connection, community and social environment; (3) practical management of MS in the wider society—policy and government regulation; and (4) global events that greatly upheave the MS journey—natural disasters and societal conflicts, such as a pandemic. These themes highlighted the complex interrelationship between socioecological factors and self‐management abilities in people living with MS.

**Conclusions:**

The diagnostic experience of those with MS is highly complex. Although it varies for each person living with MS, there are shared experiences that often reflect a common cycle of grief. An MS diagnosis provides an opportunity for self‐rediscovery, which can both influence and be influenced by socioecological factors. The social and technical nature of self‐managing MS strongly shapes the diagnostic experience, underpinning many aspects of daily living, social interaction and physical and psychological well‐being.

**Patient or Public Contribution:**

The research team worked closely with an MS‐specific consumer panel for the study design. This project was raised with this group, and preliminary results were shared at a national conference for MS during a lived‐experience consumer stream to gain additional insights.

## 1 Introduction

Multiple sclerosis (MS) is an immune‐mediated, neurodegenerative disorder that currently has no cure [1]. It is estimated that 2.9 million people worldwide live with MS, making it the most disabling neurological disease of young adults. MS management requires immunomodulating therapies to slow the progression of the disease, steroids during periods of relapse and symptomatic treatments, as needed. In addition, people living with MS (plwMS) need to be equipped with relevant resources and skills to engage in self‐management practices that help to improve their symptom burden [2, 3].

Self‐management is defined as the day‐to‐day oversight of a chronic condition, managed by the individuals living with a condition, rather than by their healthcare providers [4]. For MS, this includes managing a range of symptoms including, but not limited to, constipation, pain or neuropathy, often using existing or new health behaviour strategies aimed to improve quality of life. These behaviours may include changes in physical activity levels, stress management strategies or improved eating patterns, which plwMS are particularly interested in as a means to control and improve their disease course. In support of this, healthcare professionals need to consider factors that may help or hinder the self‐management experiences of plwMS, particularly among those newly diagnosed, as engagement with new health behaviour strategies is most probable [5].

The period surrounding an MS diagnosis is described as distressing, confusing and frustrating for both plwMS and their families [6]. The support and information provided by healthcare professionals at this critical stage of the disease are often inadequate and overly generic [6]. This may be influenced by factors such as readiness for change, organisational structures and government funding schemes [7], which can shape individuals' perceptions of MS and their relationships with healthcare professionals. These examples reflect the constructs and multiple levels of the socioecological model (SEM) [8], which considers the interrelationship between individual, interpersonal, organisational, community and public policy levels and their impact on health behaviours [9]. The SEM has been shown as an effective tool in healthcare, used to map factors affecting the diagnostic experience and to improve self‐management adherence [10]. In relation to health, this model recognises the factors that affect and are affected, with recent reviews demonstrating self‐efficacy and accessibility to be driving factors behind behaviour change for plwMS [9].

When individuals receive a new diagnosis of MS, their responses can vary significantly. Although it is common for some to pursue and adopt ‘health‐related’ behaviours to feel a sense of control over their diagnosis, others diverge from this path. For example, Russell et al. reported that plwMS reporting greater symptoms of anxiety, depression and fatigue tend to increase their intake of convenience and discretionary foods [2]. This dietary change, as a coping mechanism, suggests a shift from ‘societal ideals’ of alignment with nutrition guidelines. Furthermore, Arpey, Gaglioti, and Rosenbaum found that participants believed their socioeconomic status, as determined by age, sex, location, occupation and income, significantly impacts healthcare utilisation and their subsequent health outcomes [11]. These findings are also supported by Wang and Geng who found a higher socioeconomic status was associated with better physical health [12], and Simpson‐Yap et al. who found higher disability, greater fatigue and the presence of comorbidities negatively influenced adherence to dietary changes in MS [13]. These individual factors intersect with a broader socioecological context and environment, influencing the experience of self‐management practices among plwMS.

Although a general understanding of the factors involved in chronic disease management is important, it is essential to address the personal impact of a diagnosis to understand its overall effects. Previous qualitative research has provided insight into the challenges and changes that plwMS make after receiving a diagnosis of MS. Most recently, Topcu et al. explain the complex and dynamic stress and grief responses that occur as people make sense of and conceptualise their diagnosis [14]. This review also reinforces the idea that adjusting to a new diagnosis promotes engagement with coping strategies and self‐management behaviours, which can be influenced by personal, societal and environmental factors. However, further investigation is needed to understand the relationship between these factors and their socioecological impact on plwMS at or near the time of diagnosis.

Although studies are exploring the adjustment to a new diagnosis of MS in general, this is an area where studies appear to target specific goal‐setting approaches in those newly diagnosed [15, 16] or single disciplines of health in those with established MS. This is supported by a recent meta‐synthesis that identified only three studies of people newly diagnosed with MS [17], with limited to no exploration of the socioecological factors influencing behaviour change post‐diagnosis. Therefore, this study aimed to explore the broader socioecological influences, based on the constructs of the SEM, that affect the diagnostic experience and self‐management practices or behaviours around the point of diagnosis among plwMS.

## 2 Methods

Our study was reported according to the Consolidated Criteria for Reporting Qualitative Research (COREQ) checklist for collecting and analysing qualitative interview data [18]. Ethics approval was obtained by the University of Wollongong's Human Research Ethics Committee (Reference: 2019/269).

### 2.1 Methodological Orientation

We employed a qualitative methodology utilising a phenomenological approach to explore the socioecological influences on MS self‐management [19, 20]. A descriptive phenomenological approach is uniquely positioned to support this inquiry, as by definition, it describes the essence of a phenomenon through exploration of the lived experience [21]. Phenomenology is common to social science research to gain insight into people's actions and motivations and often involves a smaller sample of participation for an in‐depth exploration of identified themes [22]. This methodological orientation was deemed most appropriate to guide our study design and ensure a thorough exploration of the primary aim and objectives including *what* self‐management and behavioural changes were experienced post‐MS diagnosis and *how*.

### 2.2 Researcher Characteristics

Our research team consisted of one female student dietitian (S.M.; BNutrDiet fourth year) who moderated the interview process. The moderator had limited prior experience with qualitative methodology and was guided by three more experienced qualitative researchers and Accredited Practising Dietitians (Y.P., A.M. and O.W.). An observer (H.G.) was present during the interviews to record visual cues and nonverbal occurrences (e.g., body language and gestures) that were not captured with audio recording. No members of our research team were known to the participants; however, due to one researcher (Y.P.) having an MS diagnosis, their role was carefully managed to avoid potential biases or influences.

### 2.3 Sampling

People newly diagnosed with MS were recruited between February and June 2022 via multiple methods including the MS Australia website, newsletters from the Illawarra Health and Medical Research Institute, social media platforms including Facebook and a radio interview with the senior researcher (Y.P.). Individuals were asked to submit an expression of interest to the research team (S.M. and Y.P.). Telephone screening assessed eligibility based on the following criteria: (a) adults aged ≥ 18 years diagnosed with clinically definite MS confirmed by a neurologist within the last 12 months, (b) the ability to communicate in English (or access to a caregiver who could communicate on their behalf) and (c) willingness to participate in one online interview with a member of our research team (S.M.). If participants met the inclusion criteria and provided informed consent, data on gender, age, diagnosis data and the Patient‐Determined Disease Steps (PDDS)—a self‐reported measure of MS disability on a scale from 0 (normal, no disability) to 8 (bedridden due to MS)—were collected [23].

Theoretical thematic saturation guided the sample size of our study. Saturation was defined based on the point in data collection in which insights were exhausted and no new themes were identified at that point in time [24, 25]. Therefore, recruitment and data collection continued until thematic saturation of key themes was achieved. As a guide, previous studies with newly diagnosed plwMS have reported sample sizes of 8–25 [26, 27]. Participants were remunerated a $20AUD voucher for their involvement in our study.

### 2.4 Data Collection

Interviews were conducted online using Zoom (Zoom Video Communications Inc., 2022) between April and June 2022, lasting 30−60 min. A semi‐structured interview guide was developed in advance, guided by the SEM, to prompt discussion and reflection from participants on relevant topics (Table S1) [8]. The interview guide was pilot tested by the primary researcher (S.M.) and two senior researchers (Y.P. and A.M.) for reflexivity and relevance of the open‐ended questions and refined as appropriate [28]. All interviews were audio recorded in duplicate using Zoom and a mobile voice recorder (Samsung, 2022).

Verbatim transcription occurred immediately following each interview using Zoom. Manual editing was conducted for quality assurance by comparing the audio files with written transcripts, completed by the primary researcher (S.M.) using Microsoft Word (Microsoft Corporation, 2021). Transcripts were also inspected for data quality by a second researcher (Y.P.) to ensure that transcripts remained de‐identified to preserve anonymity. Observer notes of nonverbal cues, such as pauses, changes in tone and body language, were added to the transcripts to provide context to the written word. By integrating visual cues in the transcripts, the primary researcher (S.M.) was able to more accurately interpret the meaning behind participant quotes and expressions, providing a richer qualitative analysis. Reflective memos were employed to manage researcher bias, assumptions and personal interests and were included alongside the transcripts to deepen the analyses.

### 2.5 Data Analysis and Management

Deductive analysis, followed by inductive semantic analysis, was undertaken to understand the lived experiences of MS through continuous exploration of the discussed phenomena [22] informed by a recent (2023) scoping review conducted by our research team [9]. Firstly, a deductive analysis of interview transcripts used a discrete set of open codes to sort the data into a priori categories specified by the SEM [29]. The coding of transcripts was initially completed with the following theoretical constructs: individual, interpersonal, community, societal environment and natural environment. Secondly, an inductive, data‐driven stage was applied to compare and extract key themes from the data into narrower focused codes, selecting exemplar quotations from each transcript to conceptualise the individual experiences [30]. All coding was managed using NVivo software (QSR International Pty Ltd., 2020).

The final themes and related quotes were iteratively reviewed by the research team (O.W., S.M. and Y.P.) alongside the SEM. Analytical rigour was maintained through an audit trail generated with NVivo software (QSR International Pty Ltd., 2020, NVivo) where each extracted quote was cited back to the original transcript, alongside a review completed by a senior researcher (Y.P.) of the final themes and extracted quotes to improve the validity of the results [31]. Rigour was further enhanced, with both researchers (S.M. and Y.P.) engaging in reflexivity to examine how their biases might impact the interpretative phenomenological analysis and to ensure that participant experiences were fully heard [28, 31]. No repeated interviews were conducted.

## 3 Results

Eight (*n* = 6, 75%: female; *n* = 2, 25%: male) people diagnosed with MS within the last 12 months participated. All participants were diagnosed with relapsing‐remitting MS (RRMS). The average age at the time of the interview was 41.62 (± 9.59) years with a PDDS score ranging from 1 to 3, indicating a relatively low to moderate burden of MS disability (Table 1). Interviews averaged 45 min (± 5.28) in duration. Saturation was reached by the sixth participant; however, interviews with additional two participants were conducted to provide greater confidence in the reliability of our study findings.

**Table 1:** Participant sociodemographics.

ID	Age (years)	Sex	Marital status	State	Diagnosed (mm/yy)	PDDS scale	Education	Experience of symptoms before diagnosis^a^
01	25	F	Single	VIC	09/21	1	Not reported	< 1 year
03	40	F	Married	VIC	04/21	3	Bachelor's degree	11 years
04	34	F	Married	WA	09/21	2	Doctorate degree	< 1 year
05	58	M	Married	NSW	10/21	1	High school diploma	4 years
06	42	F	Married	VIC	09/21	3	Bachelor's degree	16 years
07	42	F	Married	SA	09/21	1	Bachelor's degree	< 1 year
08	46	M	Divorced	VIC	02/21	2	High school	4 years
09	46	F	Married	NSW	07/21	0	Bachelor's degree	1 year

Abbreviations: F, female; M, male; NSW, New South Wales; PDDS, patient‐determined disease steps; SA, South Australia; VIC, Victoria; WA, Western Australia.

^a^ Representing time from the first demyelinating event to clinically definite MS, as self‐reported by the participant.

**Figure 1:**
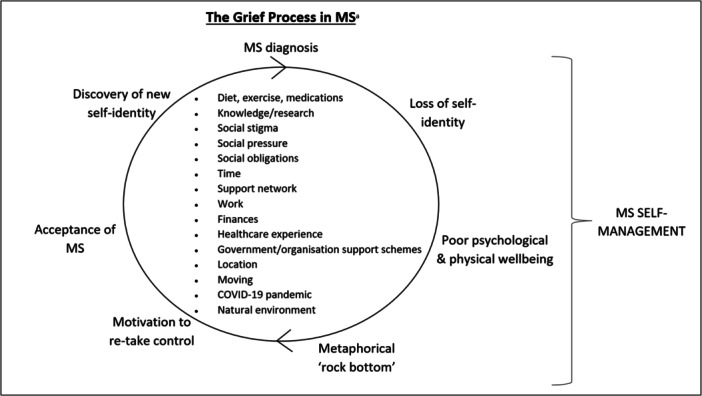
The influential factors guiding the MS self‐management journey. MS, multiple sclerosis.

Four core themes were identified: (1) taking control of a new diagnosis to retain a sense of personal identity—individual level; (2) grief and acceptance guided by community—social connection, community and social environment; (3) practical management of MS in the wider society—policy and government regulation; and (4) global events that greatly upheave the MS journey—natural disasters and societal conflicts, such as a pandemic. Most notably, these themes revealed the highly individualised reflections of each lived experience accompanied by a common feeling of loss in one's personal identity post‐diagnosis and the consequential grieving process that followed. A visual representation of the interactions between the themes is displayed in Figure 1. Although the interaction of each of the influencing factors sits at the centre of the cycle (Figure 1), it must be noted that the interaction of the factors was unique to each participant and their circumstances.

### 3.1 Theme 1: Taking Control of a New Diagnosis to Retain a Sense of Personal Identity—Individual Level

An MS diagnosis initiated a complex pathway into the grief cycle [32], following an initial sense of loss of one's identity. Many participants recounted the subtle onset of symptoms experienced over many years, which were initially mistaken for other health conditions. The endless cycle of experiencing deteriorating health while receiving no answers was influenced by individual characteristics and coping skills, such as emotions of fear, anxiety, stress and depression. Poor psychological well‐being clouded views of personal identity as doubt around personal ability became overwhelming, alongside fear of what MS now meant for their lives. These individual and emotional changes were evident as the first level from the SEM to accompany the MS diagnosis as plwMS adjusted their lifestyles.

A confronting area for participants to discuss was their identity as parents. When interview discussions explored the impact of MS on parenthood, a strong emotional response was elicited. Descriptions were heard of the experience of loss in an area that these participants felt was core to their identity and purpose.

P03: I've just realised recently that I haven't really had time to process what this means, and I think finding that balance of, [pause] what can I do now to live as well as I can for as long as I can and not to go too far down that rabbit hole of ‘what if?’ … having to support my kids to understand, I can't play with you like I used to be able to, [voice strained] I can't do those things like I used to be able to … We spoke to them [children] and tried to explain what this might mean and what might be different now but I don't look any different to them.

Furthermore, new limitations of MS rendering individuals incapable of meeting their expectations emphasised a ‘loss’ in personal identity and consequential poor psychological well‐being. As a result, participant motivation and engagement in physical well‐being strategies such as diet and exercise varied, as influenced by poor mental health and feeling a sense of defeat. As mental health improved, participants described a refusal to let their diagnosis sway their identity. In a bid to regain control participants embarked on a journey of self‐education.

P05: I did what I guess a lot of people did initially … I trolled the net to try and find out what I can do because I'm not the kind of person that likes to be out of control of things like this. I went pretty hardcore and researched the shit out of everything … this has utterly consumed my conscious hours [voice wavering], trying to find a way to navigate through this to try and buy myself some sort of a hope for the future.

Despite efforts of self‐education, participants were disappointed as they found dietary and lifestyle evidence‐based information about MS to be difficult to access, contradictory to the message(s) received from their healthcare professional and/or diluted with misinformation. They also reported feeling frustrated by a perceived lack of knowledge shared by healthcare professionals.

P04: The research isn't there and also because all the diets are contradictory … there is not so much direction from the neurologist about food or anything like that … When I started looking into the, I don't know, six or seven different diets there are for MS, that was overwhelming because one's like ‘don't eat oats’ and I'm like ‘I eat oats everyday’, one's like ‘don't eat lentils’ and I'm like ‘I eat lentils everyday’ [nervous laugh], and so I was like ‘oh my goodness my diet needs to completely change and I already have a healthy diet’. So, it was really … confronting.

Participants described the behaviour changes they sought through diet, exercise and medication regimens as the most important to regain control and self‐identity, with the concept of a ‘healthy body’ as a primary endpoint for their future selves. The diet became a core method of implementing change. With no clear evidence‐based answers around the ‘best’ diet for MS, participants found freedom in personalising and controlling their diet to their desired level, an important way of coping with the diagnosis.

P05: A combination of saturated fat control, no dairy, no eggs, no red meat, lots of veg, little bit of lean chicken, plus the intermittent fasting, and I'm probably going to start taking some supplements on top of that … hopefully I can get myself, get my body in as good a position as, and as low an inflammatory state, as I can.

### 3.2 Theme 2: Grief and Acceptance Guided by Community—Social Connection, Community and Social Environment

After diagnosis, self‐identify was commonly tied to broader societal and community influences of the SEM. Social support networks were seen to have a dual role: (a) they negatively impacted participants through social stigma and pressure, and (b) they positively impacted participants by providing a key support network. These effects were interrelated with the individual characteristics described under theme 1, leading to either worsening psychological well‐being or a nurtured acceptance post‐diagnosis.

Firstly, the compounding pressure to meet not only expectations of self but also expectations of the surrounding community was described as placing stress on the individual.

P03: I feel like it's a full‐time job focusing on all my health stuff: appointments, going to the neurologists, MRIs [magnetic resonance imaging], physio [physiotherapy], hydro [hydrotherapy], following up on other things I need to do plus remembering all of the things I need to do for the families I support, plus my own family, [laughs] plus extended family. I just had to make peace with the fact that I can't do it all and whatever I can invest in myself now is actually for myself and my family in the future.

Many negative thoughts around the MS diagnosis were influenced by preexisting stigmatisation of disability in society. Many participants discussed this stigma in relation to their working lives, with one participant recounting a fear they faced around losing employment after disclosing their diagnosis to their employer.

P01: It was meant to be a four‐month contract where they were going to extend it … in early December, I ended up telling them about my MS diagnosis which is still pretty fresh for me and then the next week … they told me that they only wanted to hire me back on part time end come January because they didn't know what would happen with my MS.

Work and income were both defined by their importance in supporting family or others close in their social network. The social stigma towards disability and the fear of losing one's career was perceived as a direct threat to participants' social networks and their sense of self, a catalyst to the grieving process described earlier.

Another area of social stigma that repeatedly resurfaced was the social pressure experienced around dietary management. Some participants described how new dietary restrictions or physical limitations, such as fatigue, acted as a barrier to participation in social events. This caused a feeling of disconnect from social circles and placed stress on feeling accepted by the wider community.

P04: The biggest impact, 100%, has been my husband's family. I think they're grieving [laughs]. They're grieving what I could and couldn't eat. Food is so important in their culture … Sunday lunch, everything's on the table … they love just basically all the things that I was avoiding … it does impact how included I feel in the group and I also feel a bit judged as well … I was joining some friends for dinner and before I'd even sat down at the table they were like, ‘Well, are you drinking? Why aren't you drinking? How long is that lasting? Is this a temporary thing? Is this a long‐term thing?’ … I've never been self‐conscious before but all of a sudden, I was self‐conscious in my group of friends and I think that's a new thing.

As mentioned earlier, although some communities were seen to negatively impact the MS journey, others were reported to positively support a path to acceptance of an MS diagnosis. Participants described friends, family, colleagues and MS groups as key to prompting them to seek help and support.

P07: My friend rang me and she said, ‘are you still having that hand‐thing? And is your face still numb?’, and I said, ‘yeah’, and she goes, ‘but have you gotten into the neurologist yet?’ and I'm like, ‘no I haven't rang them yet’, … and she's like, ‘I'm going to hang up, you're going to ring them straight‐away and then I'm going to ring them back in 15‐minutes and find out when you've got your appointment’, … so I got in the next morning.

Support networks helped participants come to an acceptance of their diagnosis and had a primary role in keeping participants grounded throughout their early MS journey.

P09: Hubby [husband] has been very supportive. Also, very grounding because for a little while there I think it was almost that I became overprotective of myself, I can't do this, and then he turned around and had gone, ‘Like hell, you've always been able to do this why can't you not do it now?’ So, he's been able to keep that balance.

This included support from community MS groups, which helped to validate some participants' experiences with MS. These online groups created safe, stigma‐free spaces where plwMS could share their struggles or improvements and helped to encourage acceptance of their new diagnosis.

P06: I've joined a couple of those forums and there's lots of questions, people will say ‘Well, I'm having this, has anyone else had it before?’ and it's just a nice feeling to know that you're not on your own.

### 3.3 Theme 3: Practical Management of MS in the Wider Society—Policy and Government Regulation

An MS diagnosis has many socioecological implications stemming from both individual and societal perspectives. However, as evident from our study, community impacts may also stem from intrapersonal factors. Participants reported adjusting to their roles or seeking new jobs, such as changing from being self‐employed to joining a company or organisation. Financial security, such as having paid sick leave, became a new priority for participants post‐diagnosis, especially those with greater social responsibilities such as supporting a family. The pressure of finances was reported by many as a cause of stress, having widespread effects on individual's ability to manage their MS.

P07: It was probably all the problems and the MS, I realised that I needed to readjust things a little bit. So, one of my oldest clients of 14 years, they ended up wanting to get a marketing manager, and then they said that, ‘we actually want it to be you because you're always telling us all the things that we should be doing, so we want you to do it’. But I actually negotiated to be working for him rather than sub‐contracting because I thought, with MS, it's probably a time in my life where I do need security in a job. So, I do need sick‐leave and holidays and a steady income.

With financial pressures posing a clear barrier to effective behaviour change and MS management, the Australian government has in place financial policies that may influence the way people navigate the help they need. Examples discussed by participants included the pharmaceutical benefits scheme (PBS), national disability insurance scheme (NDIS), universal healthcare insurance schemes (Medicare) and workers' compensation schemes (Workcover). When prompted about the use of these services during the interviews, participants spoke of how vital these policies were in relieving additional psychological stress and pressure.

P09: My medication would normally, I think it was about $64,000, however, at some point…this particular medication was approved on the PBS [pharmaceutical benefits scheme] and so it ends up costing about $80, for a dosage…prior to all of this, because I don't have things like Workcover, we'd automatically taken out income‐protection and life‐insurance…that's always been in place and we're very thankful that that's in place now because if we ever did want to increase it they're not going to do that knowing the diagnosis of MS.

Despite this government ‘safety‐net’ being in place, many participants recounted difficulties in navigating the Australian healthcare system to access additional supports for their MS. One participant described the lack of empathy and understanding they received throughout their diagnostic and management process of MS.

P03: The way that news was delivered, I think there are unfortunately many specialists who have been in the role for a really long time and obviously they have to compartmentalise and protect themselves but that humaneness and that delivery of that news just wasn't there either … it was very cold, it was very blunt, didn't actually tell me anything about what I could expect for the future, how it might impact me now, [voice wavering] how it might impact my family.

Another participant described a lack of advice and knowledge given by healthcare professionals in relation to MS. These negative experiences created a feeling of distrust towards the medical advice provided and feelings of apprehension becoming a barrier to accessibility.

P06: While we were thinking about how we're going to apply to NDIS [national disability insurance scheme] … the GP [general practitioner] and the nurses all have a meeting with you to talk about what your needs are. I almost felt like they had no idea what I actually needed so I was really surprised at their lack of knowledge, or what I felt was their lack of knowledge.

### 3.4 Theme 4: Global Events That Greatly Upheave the MS Journey—Natural Disasters and Societal Conflicts, Such as a Pandemic

The outermost layer of the SEM reflects societal influences including environmental forces and international conflicts, which were found to have extensive and far‐reaching impacts across all levels of MS self‐management post‐diagnosis. These events included natural disasters/weather‐related events and the COVID‐19 pandemic. These events were reported to diminish the little control that individuals already felt they had, disrupting the post‐diagnosis MS pathway to acceptance and effective self‐management.

As evident from our interviews, these events had impacts extending across all levels of the SEM, from triggering MS symptoms and restricting social networks to entirely changing the healthcare experience of those with MS. One participant discussed their recent move overseas and recounted how this event uprooted the social support structures they had built and left behind in Australia, alongside displacing their daily routine for an extended period.

P01: I think maybe the stress of moving is also a big thing. You know how life stressors can be such a trigger for autoimmune things?

MODERATOR: Do you feel it [moving] flares up your symptoms?

P01:Yeah, I didn't realise to the extent, but yeah…since coming back here, I've only just now moved into my actual house, and so I have a kitchen that I haven't had for two weeks. I've been eating not the best kind of foods, a lot of takeaway [embarrassed laugh] … and I definitely notice that it has made a huge impact.

MODERATOR: Not having all your friends around you, has that influenced how anxious or stressed you feel as well?

P01: Yeah, definitely. I guess I'm someone that's used to having a lot of friends around and, especially because … they were like my family, you know? So, not having that now, it's difficult.

The wider environment was also reported as an influential event and seen to influence daily life choices, adding new levels of stress and uncertainty. Extremes in environmental temperature dictated by weather (i.e., excessively hot or cold) were reported to flare up symptoms such as fatigue. One participant recounted difficulty going to work from the uncertainty in their ability to access a parking space in the shade, and their desire to apply for the organisational support scheme Australian Council for Rehabilitation of Disabled (ACROD) permit to reserve a parking space.

P04: The hardest thing was summer, and I've had a tricky time with car parking and I'll probably have to apply for an ACROD [Australian Council for Rehabilitation of Disabled] permit so that I can be guaranteed a spot in the shade … I just can't get in the car and drive without knowing that I'll be undercover … I generally haven't been doing anything social with anyone at work because it has been outside in the heat … it's just, over summer I feel like I can't commit to anything.

The disruptions caused by the global COVID‐19 pandemic were mentioned in all interviews and seen to alter the healthcare experience and MS journey. The lockdown periods across Australia caused extended separation from family and friends impacting social support and created a general fear of the virus, adding an additional layer of stress and pressure to psychological well‐being. One participant described the impact of the pandemic on their MS journey as being isolated from family and friends.

P03: When I was diagnosed neither of our families could come support us because of COVID which has made that even harder … they were like ‘we want to be there but we don't know what to do’ … even social connections, I wasn't able to go and spend time with friends and have that support through that grief and loss.

Another participant described how the lockdown impacted their treatment plan and disease management course. Their neurologist made the choice to implement an alternative medication to reduce the risk of the participant becoming immunocompromised during the uncertainty of a pandemic.

P07: I went on high‐level alert, he [doctor] said, ‘Look, because we're in the middle of a pandemic, the drug I'd need to give you makes you completely immunocompromised’, so he says, ‘we're in a rock and a hard position here … if we weren't in a pandemic, I would put you on this medication tomorrow’.

## 4 Discussion

To our knowledge, this qualitative study is the first to explore the socioecological influences impacting the diagnostic experience and self‐management practices following an MS diagnosis. The MS journey, as described by our participants, is a highly complex and individualised process, which follows the Kubler‐Ross cycle of grief [33], fuelled by a loss of personal identity, deteriorating psychological and physical well‐being, and social stigma that may challenge one's perception of self. Growing tiresome of this feeling of ‘loss’, participants described a newfound motivation to take control over their diagnosis. Key supports including healthcare professionals, social and community groups and national policies and schemes were seen to facilitate the acceptance of an MS diagnosis, underscoring the need for multifaceted interventions that address socioecological factors at the various levels. Future studies may benefit from exploring the interactions between Kubler‐Ross' cycle of grief with levels of the SEM within MS care to enhance patient outcomes.

Following an MS diagnosis, an initial sense of loss of identity sparked a desire to regain control through health‐behaviour changes to preserve and improve physical and psychological well‐being. This finding, although reflected in literature for other autoimmune conditions [34], has also been described among plwMS where the impact and fear of a new diagnosis of MS have been described to challenge self‐perception and perceived identity [17]. These intrapersonal and psychological changes to self‐identity post‐diagnosis appeared key to the beginning of what is commonly referred to in this manuscript as the MS ‘journey’. Interestingly, Schiavon et al. investigated the role of hope in chronic disease treatment, suggesting that individuals with higher levels of optimism and hope were more likely to seek engagement in health behaviours [35]. These factors can have a positive influence on psychological well‐being and may be key drivers in pursuing self‐management strategies. Our study has validated these findings, and future studies should continue to explore their influence on MS self‐management and health behaviours.

A second key finding was the dual influence of social support networks and their effects on psychological well‐being. Some participants reported social stigmas and pressures that negatively influenced their well‐being, whereas some described how social networks were key support drivers. These findings are consistent with what has been previously reported among plwMS. Similarly, Homayuni et al. found social supports, considering an appropriate response to MS, are an important facilitator that affects the quality of life, where social support is provided in the form of emotional, mental, instrumental, information and financial support [36]. Family members are often regarded as the most prominent figure to provide this support, which is often seen as a foundation in creating an environment conducive to effective self‐management of many chronic conditions [37]. In contrast, the experience of negative prejudice, judgement and exclusion as a result of society (i.e., stigmatisation of disability) are also significantly and negatively related to overall quality of life. Results from a cross‐sectional study by Kalantari et al. showed that the negative impacts of social stigma caused many plwMS to hide their disease to maintain a job, social relationships and due to the fear of a negative reaction from others in society [38]. From this, it is evident that influences of social networks can occur on a spectrum and have far‐reaching impacts, including physical and psychological influences. Future studies should aim to explore not only the perspective of lived experience but also those from a range of community and social stakeholders that may influence the physical and psychological outcomes.

The importance of social safety‐nets provided by larger work‐related organisations was underscored by the impact(s) of financial‐related stress on MS self‐management. This stress was reported to act as a barrier towards implementing behavioural changes for effective MS management, which has been reported in other qualitative literature, describing concerns about economic adjustment predominantly in the minds of those newly diagnosed with MS [39]. Given MS is a chronic disease, necessitating costly bi‐yearly and even ad hoc appointments with several healthcare professionals, these findings were somewhat expected given the most recent national, economic impacts report for MS. This report found an alarming 276% increase in annual per person costs for plwMS with no to severe disability, driven largely by costs of therapies, loss of wages and decreased informal care costs [40]. Our results demonstrate that the financial strain of MS is likely to have an impact on multiple socioecological levels, including the psychological adjustment and financial burden related to a diagnosis of MS. Therefore, addressing the financial‐related stress as reported by participants in our study is crucial to improving the overall well‐being and engagement with self‐management among plwMS.

Another significant barrier to behaviour change was a reported trust/mistrust between plwMS and healthcare professionals, which has been previously recognised as a key social determinant of health in MS [41]. Although not only common to just MS, Hudon et al. explored this notion in chronic disease management more broadly and emphasised the importance of a foundational trusting relationship between healthcare provider–patients [42]. A positive experience in healthcare appears to be highly influential towards improving self‐management strategies in MS and requires MS healthcare teams to recognise and address prior negative healthcare experiences and advocate for and support all of their patients [41]. Additionally, the individual experience within the healthcare system, especially the relationships built with healthcare professionals, is vital for clear navigation of and access to MS‐related services for effective MS self‐management [42].

Unsurprisingly, the impact of global events, most notably the COVID‐19 pandemic, was reported to impact all levels of the SEM including the MS journey post‐diagnosis for all participants. The notion of how a chronic disease diagnosis may impact major life decisions has been explored in previous studies among plwMS with influential evidence of the impact of COVID‐19 on medical appointments and medication compliance [43, 44]. By losing a sense of control, and the instilled fear of COVID‐19 for its unknown effects on MS, negative impacts on all phases of the MS journey were reflected in our study. Further research should explore longer term impacts of global events such as the COVID‐19 pandemic on plwMS, and how they employ coping and resilience mechanisms.

Our study gathered perspectives from varied lived experiences, including but not limited to different social circumstances, financial security and access to healthcare, to best represent the true characteristics and chronic disease nature of MS. Although our participants were all diagnosed within 12 months of the study, the criteria for diagnosis of MS means that some people were likely living with MS for many years before their formal clinical diagnosis. Although this is a limitation to our study, the complexity of the condition required us to consider the point of diagnosis as a marker for the journey that our participants were on to share their lived experiences. Future research with participants of more long‐standing and progressive diseases will help to explore the long‐term implications of this process, enhancing our understanding of MS self‐management across the entire disease course and SEM levels.

Although qualitative methodology emphasises an information‐rich sample, a wider range of participants may also be beneficial to the generalisability of the study findings. A second limitation was the lack of investigation into potential comorbidities that may have influenced factors such as existing familiarity with the healthcare system or financial aid. It is also worthwhile acknowledging that our interviews were conducted during the COVID‐19 pandemic; therefore, results may be heightened and not be reflective of life after the pandemic. Finally, data on ethnic group representation including race were not collected; therefore, researchers should use caution when generalising findings to reflect the diversity of the Australian population.

## 5 Conclusion

The findings of our study highlight the complexity of the MS journey post‐diagnosis, where engagement with MS self‐management behaviours is influenced by multiple levels of the SEM and their interactions. Although MS care is constantly evolving and these experiences are likely to differ between people, a core driver to the MS journey appears to be the rediscovery of self through the challenges faced post‐diagnosis. Health behaviours are largely affected by socioecological influences, which underpin many aspects of daily living, social interaction and physical and psychological well‐being.

## Author Contributions

**Olivia Wills:** conceptualisation, writing–review and editing, validation, supervision, methodology. **Sarah Manche:** conceptualisation, investigation, writing–original draft, methodology, visualisation, writing–review and editing, formal analysis, project administration. **Yasmine Probst:** conceptualisation, methodology, investigation, validation, supervision, writing–original draft, writing–review and editing.

## Ethics Statement

This study was approved by the University of Wollongong Human Research Ethics Committee (Reference: 2019/269). Written informed consent was provided by all participants before the interview, and all personal information was de‐identified and kept confidential. Any identifiers were re‐coded using a pseudonym to anonymise the collected data.

## Consent

Participants consented to the use of their data and de‐identified exemplar quotes for publication.

## Conflicts of Interest

Olivia Wills is currently receiving a post‐graduate scholarship from MS Australia. Yasmine Probst has received research grants from Multiple Sclerosis Research Australia, a fellowship from Multiple Sclerosis Australia. Yasmine Probst is a reviewer for a range of multiple sclerosis scientific journals, a member of the Multiple Sclerosis Australia grant review panel and conference committee and has received honoraria from Multiple Sclerosis Research Australia and Multiple Sclerosis Plus. Yasmine is also a person living with MS. To minimise potential bias of personal connection to the research, Yasmine was distanced from the data analysis phase. The other authors declare no conflicts of interest.

## Supporting information

Supporting information.

## Data Availability

The data that support the findings of this study are available from the corresponding author upon reasonable request.
