# Supplementary material for: A Qualitative Exploration of the Socioecological Influences Shaping the Diagnostic Experience and Self‐Management Practices Among People Newly Diagnosed With Multiple Sclerosis
Source: Health Expect. 2024 Nov 6;27(6):e70091. doi: 10.1111/hex.70091 (PMC11540932; doi:10.1111/hex.70091)
Supplement: Supplementary file 1 — Supporting information. [file HEX-27-e70091-s001.docx]

**Table S1.** Deductive framework to code interview transcripts.

| **Open Codes** | **Interview Excerpts** | **Focused Codes** |
| --- | --- | --- |
| 1. Individual  - Lifestyle (diet, exercise, stress)  - Medications  - Parenthood  - Personal identity  - Symptoms  2. Interpersonal  - Family influence  - Friend influence  - MS groups  - Partner influence  3. Community  - Health information  - Social stigma  - Work  4. Societal Environment  - Diagnosis process  - Health costs  - Healthcare experience  - NDIS scheme  5. Natural Environment  - COVID-19 pandemic  - Location  - Moving | *Question:* could you take me through your personal MS journey from around your time of diagnosis?  *Answer:* being a busy mom, working four days a week and all the other things in life it's, I’ve just realized recently that I haven't really had time to process what this means, and I think finding that balance of, what can I do now to live as well as I can for as long as I can and not to go too far down that rabbit hole of ‘what if?’…having to support my kids to understand I can't play with you like I used to be able to, I can't do those things like I used to be able to.  *Question:* How does you network of friends and family then support you during these times?  *Answer:* I did find it really hard initially to be walking around and doing our grocery shopping because I used to do that exclusively for our family and then there was the challenge of me being immunocompromised. So, my husband and I decided that he would do the shopping from now on…so I was really grateful for his support with that.  *Question:* Do you feel the government provides good accessibility to services?  *Answer:* navigating the health system, having to advocate for myself, something I'm really great at doing for families I work with but now I’m having to do it for myself. So grateful to have those skills, have that voice and know how to push back when I need to. But I think COVID has been a big challenge I would have fallen through the cracks multiple times.  *Question:* How has your diagnosis impacted on your work-life?  *Answer:* I'd actually just started a new job in the January before I was diagnosed and so I had taken six months leave from my usual role to go and try this new job and was really loving it, and then all this happened and I went ‘I don't think I can fulfill this role as I had planned’ because I was having to take so much time off.  *Question:* How has the pandemic impacted your MS journey?  *Answer:* because of the pandemic I was away from my husband and my kids for the first time actually since they'd been born, really. My husband had to do everything, he had no supports and I was in hospital for four days…my daughter, she's very young, had never been apart from me and she found that really hard. It's quite triggering for her now anytime I mentioned any appointments to her, she's like ‘are you going overnight? How long will you be away? Where are you going? What's happening?’. | 1. Taking control of a new diagnosis to retain one’s personal identity  - A sense of loss and motivation to change  - Sourcing information (or lack thereof)  2. Grief and acceptance guided by community  - Facing social pressure and stigma  - Importance of a strong support network  3. Practical management of MS in the wider society  - Accessibility and experience of the healthcare system  - Interruptions to work  4. Global events that greatly upheave the MS journey  - COVID-19 pandemic  - The natural environment |
